# Hamstring autograft, bone‐patellar‐tendon‐bone autograft and synthetic graft in primary anterior cruciate ligament reconstruction: A meta‐analysis of comparative studies

**DOI:** 10.1002/jeo2.70326

**Published:** 2025-07-18

**Authors:** Michele Mercurio, Erminia Cofano, Orlando Cosentino, Katia Corona, Fabrizio Mocini, Umberto Rossi, Olimpio Galasso, Giorgio Gasparini, Simone Cerciello

**Affiliations:** ^1^ Department of Orthopaedic and Trauma Surgery “Magna Graecia” University, “Mater Domini” University Hospital Catanzaro Italy; ^2^ Research Center on Musculoskeletal Health, MusculoSkeletal Health@UMG Magna Graecia University Catanzaro Italy; ^3^ Department of Medicine and Health Sciences “Vincenzo Tiberio” University of Molise Campobasso Italy; ^4^ Casa di Cura Villa Betania Rome Italy; ^5^ Department of Orthopaedics and Traumatology Fondazione Policlinico Universitario A. Gemelli IRCCS ‐ Sacred Heart Catholic University Rome Italy; ^6^ Department of Medicine, Surgery and Dentistry University of Salerno Baronissi Italy; ^7^ Department of Life Sciences, Health and Health Professions Link Campus University Rome Italy

**Keywords:** anterior cruciate ligament, anterior cruciate ligament reconstruction, autograft, bone patellar tendon bone graft, graft failure, hamstring, synthetic graft

## Abstract

**Purpose:**

Anterior cruciate ligament reconstruction (ACLR) yields favourable results, but failure and reinjury rates are still a concern. Graft choice is a modifiable risk factor for surgeons to avoid failure. The topic of optimal graft selection remains a subject of ongoing debate. Graft choices include autografts, allografts and synthetic grafts. The purpose of this meta‐analysis was to compare functional outcomes and complications between autografts, hamstring (HT) tendon and bone‐patellar‐tendon‐bone, and synthetic graft in primary ACLR.

**Methods:**

The PubMed, MEDLINE, Scopus and Cochrane Central databases were used for the research, and nine studies were included. The first author, journal name, year of publication, patient demographics, type of surgery, type of graft used for ACLR, time from injury to surgery, and follow‐up period were recorded. The data extracted for quantitative analysis included Lysholm activity scale score, Tegner activity scale score, International Knee Documentation Committee (IKDC) score, laxity measured with the KT‐1000 knee arthrometer (KT‐1000), number of complications, re‐rupture, and re‐intervention rates. Random and fixed effect models were used for the meta‐analysis of pooled mean differences (MDs) and odds ratios (ORs).

**Results:**

A total of 734 patients were identified, 377 of whom underwent ACLR with autograft and 326 with synthetic graft. The mean age was 28.7 ± 20.3 and 31.6 ± 9.3 years for the ACLR with autograft and ACLR with synthetic graft groups. The mean follow‐up durations were 82.3 ± 38 and 81.4 ± 39.2 months. Comparable postoperative Lysholm knee score (*p* = 0.06), Tegner activity scale score (*p* = 0.64) and IKDC score (*p* = 0.15) were found between groups. Significantly greater knee laxity was found in the autograft group (2.6 ± 1.4 and 1.5 ± 1.4 mm; MD = 1.22, 95% confidence interval [CI]: 0.96, 1.48; *p* < 0.001). Comparable overall complications (*p* = 0.70), re‐rupture (*p* = 0.81) and re‐intervention (*p* = 0.85) rates were found between groups.

**Conclusions:**

Compared to ACLR with HT autograft, the ACLR with synthetic graft showed statistically but not clinically important decreased knee laxity. Comparable functional outcomes, complication and re‐rupture rates were found between the two groups.

**Level of Evidence:**

Level I, meta‐analysis.

**Abbreviations**
ACL: anterior cruciate ligament
ACLR: anterior cruciate ligament reconstruction
BPTB: bone‐patellar‐tendon‐bone
HT: hamstring
IKDC: International Knee Documentation Committee.

## INTRODUCTION

Anterior cruciate ligamen (ACL) reconstruction yields favourable results and recent advances in the comprehension of the anatomy and biomechanics of the ACL have led to advancements in the surgical treatment options. However, failure and reinjury rates are still a concern, especially for younger people who participate in pivoting sports [25, 39, 40]. Walden et al. reported that a large proportion of these injuries occur in the first year after ACL reconstruction (ACLR), with 4% of patients suffering graft rupture before returning to match play and an additional 3% being reinjured within 3 months after their first match appearance [38]. Many causes of graft failure have been reported in literature and graft choice is a modifiable risk factor for surgeons [28]. The topic of optimal graft selection remains a subject of ongoing debate [2]. Graft choices include autografts, allografts and synthetic grafts [22]. Autografts are the most common option because of their biological properties, costs, positive results and favourable return to high‐level sports [8, 36]. The bone‐patellar‐tendon‐bone (BPTB) autograft is considered the gold standard, later the hamstring (HT) autograft became popular for less donor site morbidity [7]. Allograft is an alternative that prevents the problems associated with graft harvest [1, 17], but concerns about disease transmission, immune reactions [12], costs and availability are reported [23]. Currently, allografts are an acceptable option in revision cases, patients not returning to pivoting sports, or patients over 40 years of age [2, 9]. Synthetic grafts became popular during the 1970s and 1980s for providing immediate tensile strength and fast rehabilitation without the risks of disease transmission and immunological rejection. They have been used to overcome stiffness and strength problems by providing tensile strength, reducing donor site morbidity, and allowing a faster return to activity, but they have shown a higher rate of failure/rupture that causes synovitis, sterile effusion and the accumulation of synthetic debris material within the knee [3].

Although many studies support one type of transplant over another, the confounding variables of such a complex procedure are often difficult to control and may affect the results [6, 33]. Several studies compared outcomes of ACLR with autograft versus synthetic graft but there is still no consensus regarding the optimal graft choice in primary ACLR.

The purpose of this meta‐analysis was to compare functional outcomes and complications between autografts, HT tendon and BPTB, and synthetic graft in primary ACLR. All available homogeneous data from comparative studies were pooled to identify differences between the graft options and to provide guidance to surgeons.

## MATERIALS AND METHODS

### Search strategy

A systematic review of the published literature was conducted, and the results are reported according to the preferred reporting items for systematic reviews and meta‐analyses (PRISMA) statement [27]. The PubMed, MEDLINE, Scopus and Cochrane Central databases were searched in April 2024, with no lower date limit. The terms ‘anterior cruciate ligament’, ‘reconstruction’, ‘autograft’, ‘synthetic graft’, ‘knee’, ‘outcome’ and ‘results’ were used in different combinations to retrieve relevant articles. The articles were selected based on the following PICO model: [35] (P) patients with ACL injury, (I) patients who underwent primary ACLR with autograft, (C) patients who underwent primary ACLR with synthetic graft, and (O) patients assessed for functional outcomes and complications.

Two authors (U.R. and M.M.) independently conducted all the searches and screened the titles and abstracts to identify articles for inclusion. If a study could not be excluded based on the title and abstract, both reviewers reviewed the full text to reach a consensus on the inclusion or exclusion of the study, contacting a third senior author (S.C.) in case of major discrepancies. The reference list of each included article and the available grey literature at our institution were screened for the inclusion of potential additional articles.

### Inclusion criteria and study selection

The inclusion criteria were applied during the title, abstract and full‐text screenings and were as follows: (1) observational studies including case‐control, cohort studies and randomised controlled trials (RCTs); (2) reporting comparative outcomes and/or complications of primary ACLR with autograft or synthetic graft; (3) reporting the data of >10 surgically treated cases; and (4) written in English. The exclusion criteria were as follows: (1) radiological study, (2) involving multiple‐ligament reconstruction and (3) involving revision surgery for the ACLR. Other reviews, case reports, cadaveric or biomechanical studies, technical notes, editorials, letters to the editor and expert opinions were excluded from the analysis but considered for the discussion section.

### Data extraction and quality assessment

Two authors (U.R. and M.M.) performed comprehensive data extraction from the included articles. The first author, journal name, year of publication, patient demographics, type of surgery, type of autograft and synthetic graft used for ACLR, time from injury to surgery, and follow‐up period were recorded [8]. The data extracted for quantitative analysis included the Lysholm activity scale, the Tegner activity scale score, the International Knee Documentation Committee (IKDC) score, laxity measured as anterior tibial motion relative to the femur with the KT‐1000 knee arthrometer (KT‐1000), number of complications, re‐rupture and re‐intervention rates.

A methodological quality assessment was conducted independently by three authors (E.C., M.M. and S.C.) with the modified Newcastle‐Ottawa Quality Assessment Scale [42] (Supporting Information S1: Table S1). The Cochrane risk of bias tool was used to evaluate the risk of bias in the included randomised controlled studies (Supporting Information S1: Tables S2 and S3).

### Data synthesis

All the data were reported with one‐decimal accuracy. The mean, standard deviation and range were noted for the continuous variables, and the count was noted for the categorical variables. Functional outcomes and complications were entered into a meta‐analysis of pooled mean differences (MDs) and odds ratios (ORs), respectively [26]. The Mantel–Haenszel method was adopted according to the Cochrane Statistical Methods Group. Random or fixed effects models were employed based on the heterogeneity of the between‐trials as calculated by the *I* ^2^ statistics; in particular, random effects models were used when considerable heterogeneity (*I* ^2^ > 50%) was noted unless the between‐studies variance (*σ* ^2^) was poor, in which case fixed effects models were used despite the heterogeneity found. When possible a further subgroup analysis was performed according to the specific type of graft used (i.e., HT or BPTB). Review Manager (RevMan 5.3, Cochrane Collaboration, Nordic Cochrane Center) was used for the statistical calculations; a *p*‐value < 0.05 was considered significant.

## RESULTS

**Figure 1:**
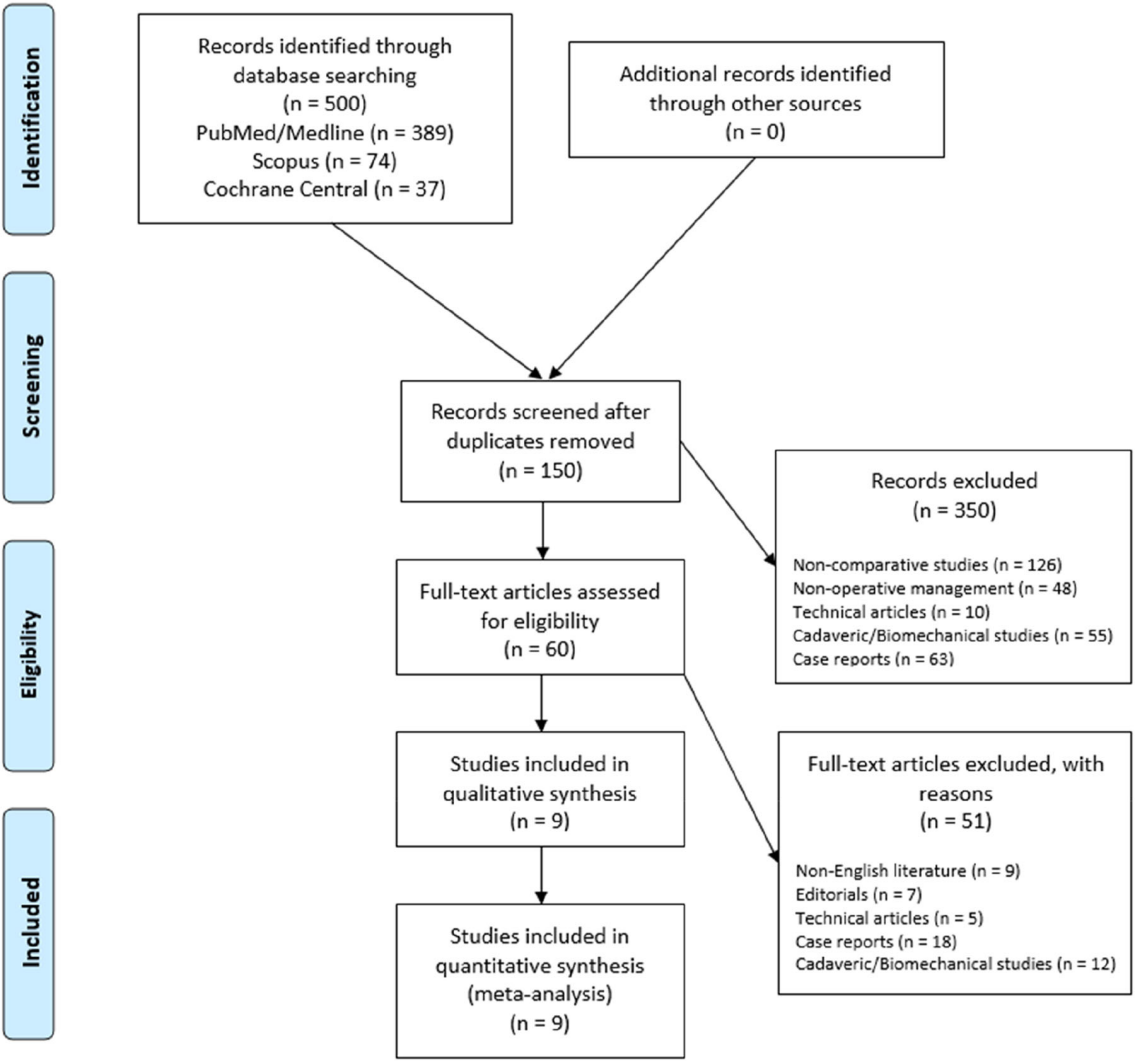
Preferred reporting items for systematic review and meta‐analysis (PRISMA) flowchart for the searching and identification of included studies. For more information, visit www.prisma-statement.org [27].

A total of 500 relevant articles were identified through the initial search, 150 abstracts were screened, and 60 full‐text articles were assessed for eligibility based on our inclusion criteria, resulting in nine comparative studies that were eligible for the meta‐analysis (Figure 1).

The included studies were published from 2009 to 2023; all studies were cohort studies, of which three were prospective and six were retrospective.

Four studies were conducted in China, two in Italy, one in Kuwait, one in the United States and one in the United Kingdom.

A total of 734 patients were initially identified, 31 of whom were lost to follow‐up, 377 of whom underwent ACLR with autograft, and 326 of whom underwent ACLR with synthetic graft. Of the 377 patients who underwent ACLR with autograft, 262 and 89 had HT and BPTB, respectively. There were 79.6% and 77.3% of male patients in the autograft and synthetic groups, respectively (Table 1).

**Table 1:** Characteristics of included studies.

Author	Journal	Year of publication	Group	Patient demographics														
				Number of patients (*N*)	Sex (*N*)		Age (years)			BMI			FU (months)			Injury to surgery (months)		
					Male	Female	Mean	SD	Range	Mean	SD	Range	Mean	SD	Range	Mean	SD	Range
Liu et al.	International Orthopaedics	2009	4SHG	32	24	8	32	9	20–56	NA	NA	NA	49	1	48–52	9	7	5–33
			LARS	28	21	7	36	9	18–54	NA	NA	NA	49	1	48–52	8	7.5	4–34
Hamido et al.	Orthopaedics & Traumatology: Surgery & Research	2015	4SHG	45	44	1	20	3.25	18–31	NA	NA	NA	59	1	58–62	8	5.7	6–29
			LARS	27	27	0	24	3.5	21–35	NA	NA	NA	59	1	58–62	7	6.2	6–31
Chen et al.	The American Journal of Sports Medicine	2017	4STG	73	64	9	28.6	8.8	17–52	NA	NA	NA	122.9	11.8	NA	18	26.1	1–208
			LARS	38	28	10	27.6	9.3	17–54	NA	NA	NA	120.8	26.9	NA	16.5	17.6	1–78
Bianchi et al.	European Journal of Orthopaedic Surgery & Traumatology	2018	4SHG	25	21	4	24	4.5	18–36	NA	NA	NA	95.4	37	85–112	NA	NA	NA
			LARS	25	20	5	37	1.5	35–41	NA	NA	NA	95.7	6	86–110	NA	NA	NA
Su et al.	Clinical Journal of Sport Medicine	2018	4SHG	67	52	15	31.1	9.75	17–56	24.5	3	18.9–31.1	84.3	17.2	60–129	NA	NA	NA
			LARS	71	48	23	32.8	11	13–57	25.8	5.7	19.1–42	96.1	18	60–132	NA	NA	NA
Moretti et al.	Advances in Orthopaedics	2023	4ST	20	20	0	35	3.6	NA	21.1	2.9	NA	36.7	8.7	NA	NA	NA	NA
			LARS	19	19	0	36.2	4.4	NA	22.5	2.6	NA	39.4	10	NA	NA	NA	NA
Pritchett et al.	Journal of Knee Surgery	2009	BPTB	35	24	11	25	6	18–42	NA	NA	NA	132	33	84–216	11	14.2	4–61
			BRAIDED SYNTHETIC	35	23	12	26	6	19–43	NA	NA	NA	144	33	96–228	14	17.5	2–72
Ghalayini et al.	The Knee	2010	BPTB	24	18	6	31	1.4	28.1–33.6	NA	NA	NA	33	13.5	6–60	33	7	19–47
			LEEDS‐KEIO	22	20	2	31.7	3.4	29–34.5	NA	NA	NA	33	13.5	6–60	55	10	35–75
Pan et al.	European Journal of Orthopaedic Surgery & Traumatology	2012	BPTB	30	19	11	34	6.3	NA	NA	NA	NA	50	1.5	48–54	13.8	8.3	NA
			LARS	32	25	7	35.9	11.3	NA	NA	NA	NA	50	1.5	48–54	11.9	7	NA

Abbreviations: ACL, anterior cruciate ligament; BMI, body mass index; BPTB, bone‐patellar to bone; FU, follow‐up; NA, not available; SD, standard deviation; SHG, hamstring tendon graft; ST, semitendinosus.

The mean age was 28.7 ± 20.3 and 31.6 ± 9.3 years for the ACLR with autograft and ACLR with synthetic graft groups, respectively (Supporting Information S1). The mean follow‐up durations were 82.3 ± 38 and 81.4 ± 39.2 months for the autograft and synthetic graft groups, respectively.

Different types of grafts, including quadrupled semitendinosus [28], four‐strand HT tendon graft [4, 7, 14, 20, 36] and BPTB [13, 32, 34] were used for the ACLR with autograft while a ligament augmentation and reconstruction system (LARS) (Surgical Implants and Devices) [4, 7, 14, 20, 28, 32, 36], a braided synthetic ligament [34], and the Leeds‐Keio (LK) [13] ligament were used for the ACLR with synthetic graft.

For an accurate analysis, we first compared the autografts group with the synthetics graft group and as further analysis we divided the group of autografts into HS and BPTB, and each of these was compared with the group of synthetic grafts.

### Time from injury to surgery

The time from injury to surgery was reported in six studies [7, 13, 14, 20, 32, 34], for 249 patients in the ACLR with autograft group and 182 patients in the ACLR with synthetic graft group, and no difference was found between the groups (13 ± 2.9 and 9.4 ± 22.2 months in the autograft and in the synthetic graft groups, respectively; MD = −3.29, 95% CI: −10.51, 3.93; *p* = 0.37) (Supporting Information S2). When analysing HT autografts only [7, 14, 20], 150 patients were available in the HT group and 93 patients in the synthetic graft group; no difference emerged between the groups (13 ± 19.4 and 11.2 ± 13.1 months in the HT autograft and in the synthetic graft groups, respectively; MD = 1.04, 95% CI: −1.16, 3.23; *p* = 0.35) (Supporting Information S3). When analysing BPTB autograft only [13, 32, 34], 89 patients were available in the BPTB group and 89 patients in the synthetic graft group; no difference was found between the groups (17.6 ± 14.2 and 29.9 ± 21.2 months in the BPTB autograft and in the synthetic graft groups, respectively; MD = −7.72, 95% CI: −23.66, 8.23; *p* = 0.34) (Supporting Information S4).

### Functional outcomes

Six studies [7, 14, 20, 32, 34, 36] reported the preoperative Lysholm knee score in 282 patients in the autograft group and 182 patients in the synthetic graft group, and no difference was found between the groups (48.5 ± 13 and 49.6 ± 14.5 for the autograft and for the synthetic graft groups, respectively; MD = −0.58, 95% CI: −1.92, 0.77; *p* = 0.40) (Supporting Information S5).

When analysing HT autografts only [7, 14, 20, 36], 217 patients were available in the HT group and 164 patients in the synthetic graft group; no difference emerged between the groups (50.9 ± 12.8 and 53.9 ± 13.5 for the HT autograft and for the synthetic graft groups, respectively; MD = −1.40, 95% CI: −2.95, 0.15; *p* = 0.08 (Supporting Information S6). When analysing the BPTB autograft only [32, 34], 65 patients were available in the BPTB group and 67 patients in the synthetic graft group; no difference was found between the groups (40.7 ± 10.3 and 39.1 ± 11 ACLR for the BPTB autograft and for the synthetic graft groups, respectively; MD = 1.91, 95% CI: −0.79, 4.62; *p* = 0.17) (Supporting Information S7).

Six studies [7, 14, 20, 32, 36] investigated the preoperative Tegner activity scale score in 247 patients in the autograft group and 196 patients in the synthetic graft group, and no difference was found between the groups (3 ± 1 and 3.2 ± 1.2 for the autograft and for the synthetic graft groups, respectively; MD = −0.15, 95% CI: −0.41, 0.12; *p* = 0.28) (Supporting Information S8).

When analysing HT autografts only [7, 14, 20, 36], 217 patients were available in the HT group and 164 patients in the synthetic group; no difference emerged between the groups (3 ± 1.1 and 2.6 ± 2.3 for the HT autograft and for the synthetic graft groups, respectively; MD = −0.02, 95% CI: −0.46, 0.43; *p* = 0.93) (Supporting Information S9).

Three studies [7, 34, 36] investigated the preoperative IKDC score in 175 patients in the autograft group and in 144 patients in the synthetic graft group, and no difference was found between the groups (49.3 ± 11.4 and 50 ± 9.4 for the autograft and for the synthetic graft groups, respectively; MD = −0.20, 95% CI: −2.89, 2.48; *p* = 0.88) (Supporting Information S10). When analysing HT autografts only [7, 36], 140 patients were available in the HT group and 109 patients in the synthetic group; no difference emerged between the groups (46.7 ± 11 and 47.2 ± 8.7 for the HT autograft and for the synthetic graft groups, respectively; MD = −0.99, 95% CI: −5.29, 3.32; *p* = 0.65) (Supporting Information S11).

**Figure 2:**
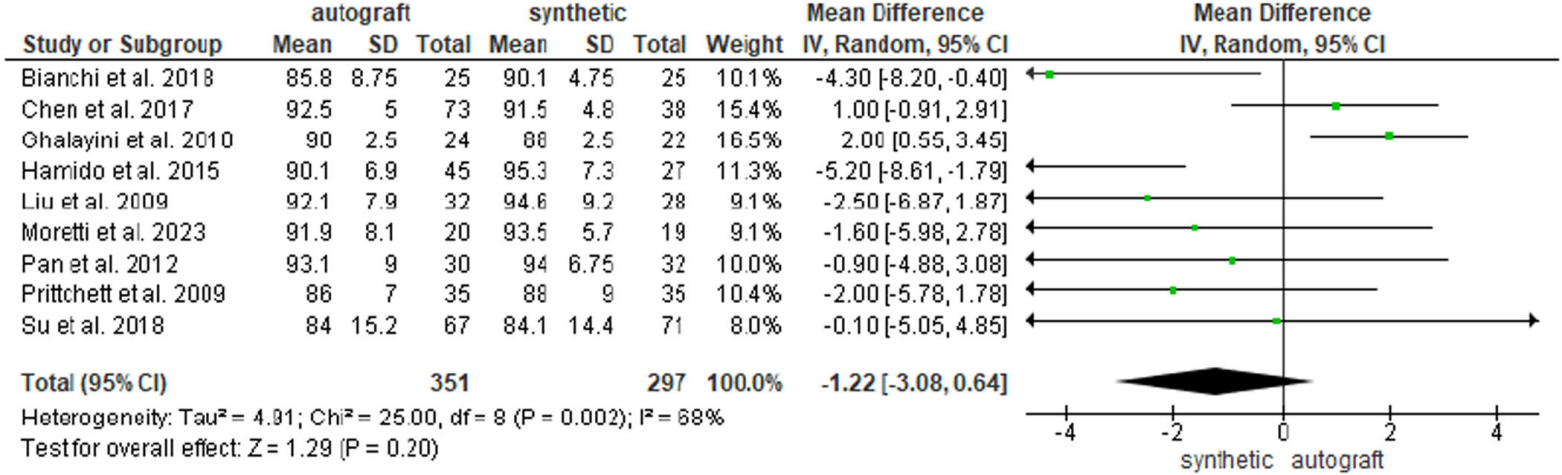
Comparison of the postoperative Lysholm activity scale between autograft and synthetic graft reconstruction groups: forest plot of effect sizes. CI, confidence interval; SD, standard deviation.

Nine studies [4, 7, 13, 14, 20, 28, 32, 34, 36] reported the postoperative Lysholm knee score in 351 patients in the autograft group and in 297 patients in the synthetic graft group, and no difference was found between the groups (89.5 ± 8.6 and 91.4 ± 8.4 for the autograft and for the synthetic graft groups, respectively; MD = −1.22, 95% CI: −3.08, 0.64; *p* = 0.20) (Figure 2).

When analysing HT autografts only [4, 7, 14, 20, 28, 36], 262 patients were available in the HT group and 208 patients in the synthetic graft group, and no difference was found between the groups (89.1 ± 9 and 91.7 ± 8 for the HT autograft and for the synthetic graft groups, respectively; MD = −2.00, 95% CI: −4.41, −0.41; *p* = 0.10. (Supporting Information S12).

When analysing BPTB autografts only [13, 32, 34], 89 patients were available in the BPTB group and 89 patients in the synthetic graft group; no difference was found between the groups (90.4 ± 7.6 and 90 ± 6.8 for the BPTB autograft and for the synthetic graft groups, respectively; MD = 0.19, 95% CI: −2.53, 2.92; *p* = 0.89) (Supporting Information S13).

**Figure 3:**
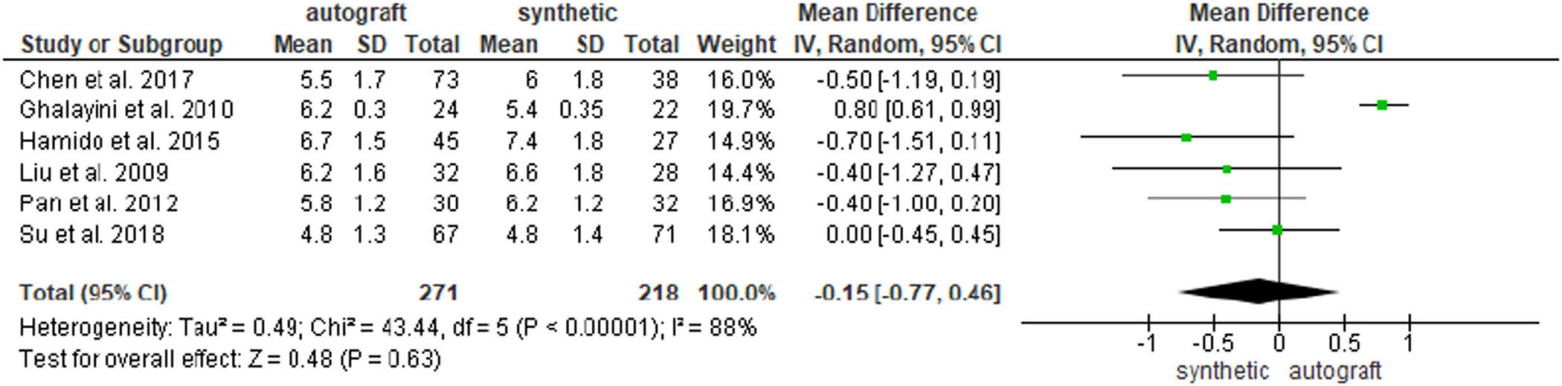
Comparison of the postoperative Tegner activity scale between autograft and synthetic graft reconstruction groups: forest plot of effect sizes. CI, confidence interval; SD, standard deviation.

Six studies [7, 13, 14, 20, 32, 36] investigated the postoperative Tegner activity scale score in 271 patients in the autograft group and in 218 patients in the synthetic graft group, and no difference was found between the groups (5.3 ± 3.3 and 6.2 ± 1.7 for the autograft and for the synthetic graft groups, respectively; MD = −0.15, 95% CI: −0.77, 0.46; *p* = 0.63) (Figure 3).

When analysing HT autografts only [7, 14, 20, 36], 217 patients were available in the HT group and 164 patients in the synthetic graft group; and no difference was found between the groups (6.1 ± 1.6 and 6.4 ± 4 for the HT autograft and for the synthetic graft groups, respectively; MD = −0.27, 95% CI: −0.59, 0.05; *p* = 0.10) (Supporting Information S14).

When analysing BPTB autografts only [13, 32], 54 patients were available in the BPTB group and 54 patients in the synthetic graft group; no difference was found between the groups (6 ± 0.9 and 5.8 ± 0.9 for the BPTB graft and for the synthetic graft groups, respectively; MD = 0.29, 95% CI: −0.79, 1.36; *p* = 0.60) (Supporting Information S15).

**Figure 4:**
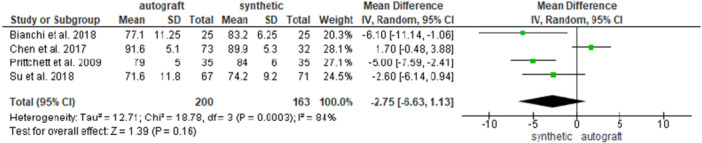
Comparison of the postoperative International Knee Documentation Committee score between autograft and synthetic graft reconstruction groups: forest plot of effect sizes. CI, confidence interval; SD, standard deviation.

Four studies [4, 7, 34, 36] investigated the postoperative IKDC score in 200 patients in the autograft group and in 163 patients in the synthetic graft group, and no difference was found between the groups (79.3 ± 11.4 and 83.3 ± 7.8 for the autograft and for the synthetic graft groups, respectively; MD = −2.75, 95% CI: −6.63, 1.13; *p* = 0.16) (Figure 4).

When analysing HT autografts only [4, 7, 36], 112 patients were available in the HT group and 115 patientsin the synthetic graft group; no difference was found between the groups (79.3 ± 12.3 and 83.1 ± 8.1 for the HT graft and for the synthetic graft groups, respectively; MD = −2.25, 95% CI: −7.98, 3.48; *p* = 0.44) (Supporting Information S16).

**Figure 5:**
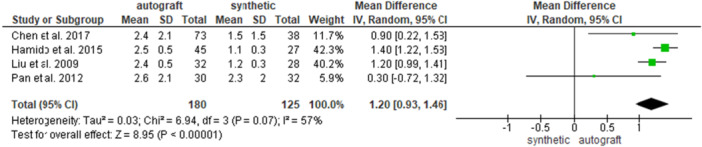
Comparison of the postoperative KT‐1000 values between autograft and synthetic graft reconstruction groups: forest plot of effect sizes. CI, confidence interval; SD, standard deviation.

Four studies [7, 14, 20, 32] investigated the postoperative KT‐1000 value, for 180 patients in the autograft group and for 125 patients in the synthetic graft group. A significantly greater knee laxity was found in the autograft group (2.6 ± 1.4 and 1.5 ± 1.4 mm for the autograft and for the synthetic graft groups, respectively; MD = 1.20, 95% CI: 0.93, 1.46; *p* < 0.00001) (Figure 5).

**Figure 6:**
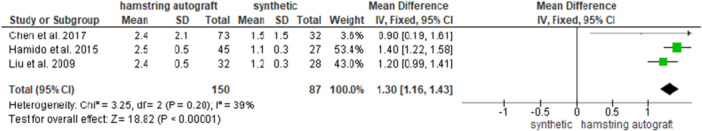
Comparison of the postoperative KT‐1000 values between hamstring autograft and synthetic graft reconstruction groups: forest plot of effect sizes. CI, confidence interval; SD, standard deviation.

When analysing HT autografts only [7, 14, 20], 150 patients were available in the HT group and 87 patients in the synthetic graft group; a significantly greater knee laxity was found in the HT autograft group (2.4 ± 1 and 1.2 ± 0.8 for the HT group and for the synthetic graft groups, respectively; MD = 1.30, 95% CI: 1.16, 1.43; *p* < 0.00001). (Figure 6).

### Complications, re‐rupture and re‐intervention

**Figure 7:**
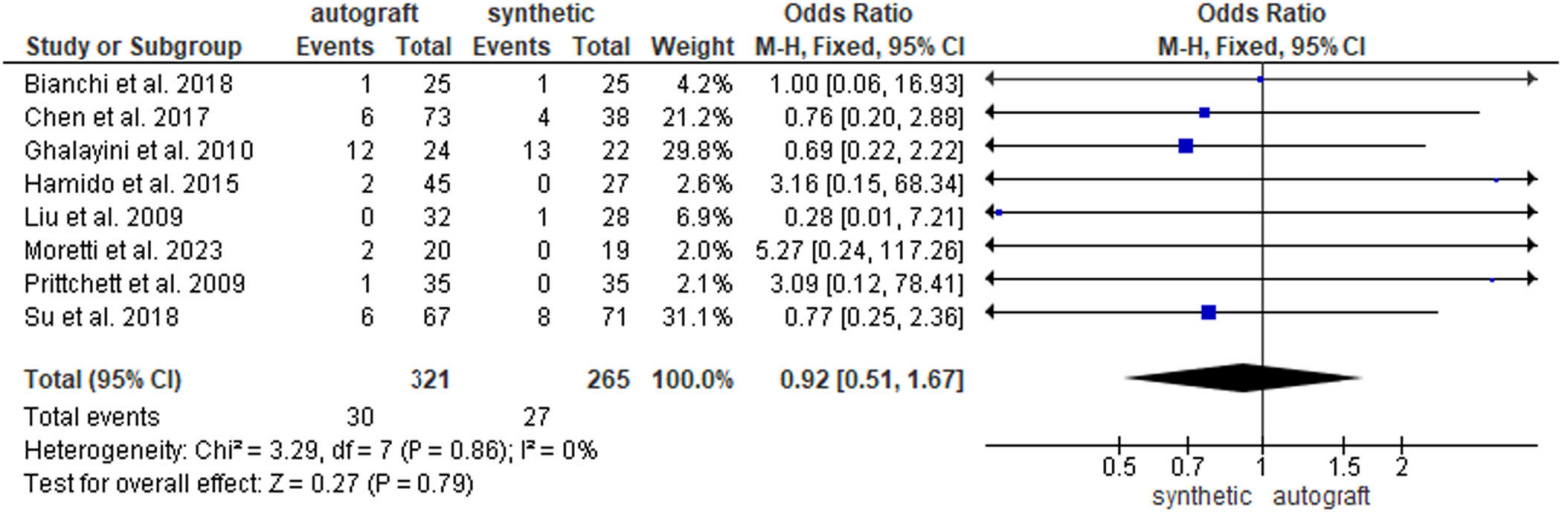
Comparison of the overall complications rate between autograft and synthetic graft reconstruction groups: forest plot of effect sizes. CI, confidence interval; SD, standard deviation.

Eight studies [4, 7, 13, 14, 20, 28, 34, 36] investigated the overall complications rate in 321 patients in the autograft group and in 265 patients in the synthetic graft group. No significant differences were found between the groups (9.3% and 10.2% for the autograft and for the synthetic graft groups, respectively; OR = 0.92, 95% CI: 0.51, 1.67; *p* = 0.79) (Figure 7).

When analysing HT autografts only [4, 7, 14, 20, 28, 36], 262 patients were available in the HT group and 208 patients in the synthetic graft group; no difference emerged between the groups (6.5% and 6.7% for the HT group and for the synthetic graft groups, respectively; OR = 0.96, 95% CI: 0.47, 1.95; *p* = 0.90) (Supporting Information S17). When analysing BPTB autografts only [13, 34], 59 patients were available in the BPTB group and 57 patients in the synthetic graft group; no difference was found between the groups (22% and 22.8% for the BPTB graft and for synthetic graft groups, respectively; OR = 0.85, 95% CI: 0.29, 2.84; *p* = 0.77) (Supporting Information S18).

**Figure 8:**
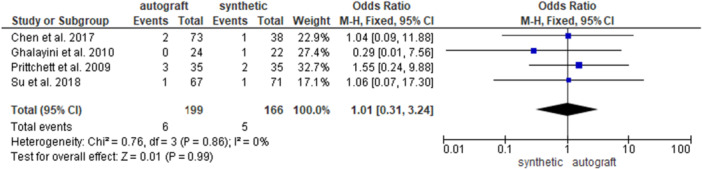
Comparison of the re‐rupture rate between autograft and synthetic graft reconstruction groups: forest plot of effect sizes. CI, confidence interval; SD, standard deviation.

Four studies [7, 13, 34, 36] investigated the re‐rupture rate in 199 patients in the autograft group and in 166 patients in the synthetic graft group. No significant differences were found between the groups (3% and 3% for the autograft and for the synthetic graft groups, respectively; OR = 1.01, 95% CI: 0.31, 3.24; *p* = 0.99) (Figure 8).

When analysing HT autografts only [7, 36], 140 patients were available in the HT group and 109 patients in the synthetic graft group; no difference emerged between the groups (2.1% and 1.8% for the HT group and for the synthetic graft groups, respectively; OR = 1.05, 95% CI: 0.17, 6.58; *p* = 0.96) (Supporting Information S19). When analysing BPTB autografts only [13, 34], 59 patients were available in the BPTB group and 57 patients in the synthetic group; no difference was found between the groups (5% and 5.3% for the BPTB group and for the synthetic graft groups, respectively; OR = 0.98, 95% CI: 0.21, 4.47; *p* = 0.97) (Supporting Information S20).

**Figure 9:**
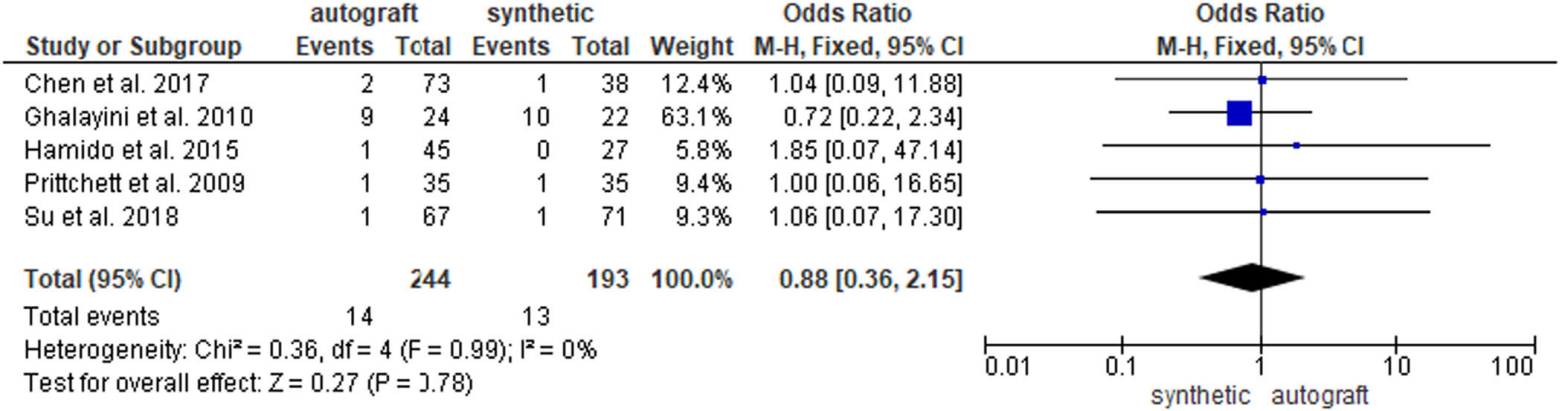
Comparison of the re‐intervention rate between autograft and synthetic graft reconstruction groups: forest plot of effect sizes. CI, confidence interval; SD, standard deviation.

Five studies [7, 13, 14, 34, 36] investigated the re‐intervention rate in 244 patients in the autograft group and in 193 patients in the synthetic graft group. No significant differences were found between the groups (5.7% and 6.7% for the autograft and for the synthetic graft groups, respectively; OR = 0.88, 95% CI: 0.36, 2.15; *p* = 0.78) (Figure 9).

When analysing HT autografts only [7, 14, 36], 185 patients were available in the HT group and 136 patients in the synthetic group; no difference emerged between the groups (2.2% and 1.5% for the HT group and for the synthetic graft groups, respectively; OR = 1.22, 95% CI: 0.25, 5.94; *p* = 0.81) (Supporting Information S21). When analysing BPTB autografts only [13, 34], 59 patients were available in the BPTB group and 57 patients in the synthetic group; no difference was found between the groups (16.9% and 19.3% for the BPTB group and for the synthetic graft groups, respectively; OR = 0.76, 95% CI: 0.26, 2.24; *p* = 0.61) (Supporting Information S22).

## DISCUSSION

The most important finding of the present study was that synthetic graft showed decreased knee laxity on KT‐1000 test when compared to autograft. However, this statistically significant difference did not lead to clinically important difference for the patients. Comparable functional outcomes, complication and re‐rupture rates were found between the groups.

ACLR still remains one of the most common procedures among orthopaedic sports medicine specialists [16, 21]. Nowadays, autograft is considered the gold standard, providing reliable long‐term results. Regardless of the type, autograft harvest can result in a degree of morbidity, which may negatively affect recovery after ACLR [19, 41]. Currently, BPTB and HT autografts are the most commonly used grafts. The intended benefits of the synthetic graft consist of avoiding donor‐site morbidity, providing a strong stabilising construct, and allowing a faster return to sport [24, 37]. Based on the findings of this study, there were no statistically significant differences between autografts and synthetic grafts for primary ACLR in the preoperative and postoperative knee functionality. These findings concur with those reported in the literature; several studies showed no difference in postoperative Lysholm scores between synthetic and autograft groups [4, 18, 37]. We also found comparable functional outcomes in terms of Lysholm and Tegner scale scores between the synthetic and HT groups. Differently, Chen et al. [7] reported that the Lyshom and Tegner scores were higher in the synthetic graft group suggesting that it was due to the early rehabilitation, no donor site morbidity and the desire to return to sport [18]. Conversely, Zeng et al. [41] found a higher Tegner score in favour of the autograft group. Su et al. [36] compared the outcomes of HT autograft and LARS in a Chinese cohort of patients, showing lower Tegner scores than those reported in European and American studies. The authors argued that the Tegner score was developed in Western countries to assess sports performance level and it might not perfectly apply to Chinese sports. According to these observations, the difference in scores between the two groups of the current study should be interpreted comprehensively and the differences were lower than the minimum clinically relevant differences reported for Lysholm [30] (i.e., 5.5 points) and Tegner [29] (i.e., 1 point) scores.

The results of the current meta‐analysis showed a decreased postoperative knee laxity as measured via the KT‐1000 arthrometer in the synthetic graft cohort. It was reported that synthetic grafts have sufficient strength due to the polyester fibre composition, ranging from 2500N to 3600N and the LARS allows high resistance to fatigue especially in the flexion‐torsion stress and in elongation [14]. Fan et al. [11] found no difference in terms of knee stability comparing BPTB autografts versus synthetic grafts. Cristiani et al. [10] concluded that HT autograft resulted in greater postoperative knee laxity compared with BPTB autograft and Nau et al. [31] found that the mean knee laxity was greater in the LARS group when compared with the BPTB group. However, it should be considered that the percentage of BPTB was low in the current meta‐analysis, and it was not possible to perform a direct comparison with the synthetic group in terms of knee stability. Moreover, the postoperative difference between the autograft and synthetic groups was less than 5 mm which is the cut‐off used to define surgical failure. These findings, therefore, need to be interpreted with caution.

In the current study, no statistically significant difference was observed in terms of complications, re‐ruptures and re‐intervention between autografts and synthetic graft. The same evidence was also observed when considering the two subgroups of autografts (HT and BPTB). Graft failure is a key outcome and it should be considered in all patients that complain of instability while playing sports. Three aspects can lead to ACL failure: surgical technical errors, graft incorporation failure and new traumas [15]. Chen et al. [7] in a 10‐year longitudinal study reported a cumulative failure rate of 8.2% and 7.9% for autograft and synthetic graft groups, respectively; the authors noted no differences between the groups, and the percentages reported are similar to those of the present meta‐analysis. The meta‐analysis by Sun et al. found that autografts were associated with a higher postoperative overall complication rate compared with synthetic grafts, but there was no significant difference between the two groups in terms of graft failure rate [37].

The findings of this study should be interpreted with respect to several limitations. First, only English studies were considered, potentially contributing to publication bias; moreover, although four recommended databases were used for the search, we cannot exclude the possibility that additional articles could have been found by searching other databases [5]. Second, in order to maintain sufficient power for the meta‐analysis, we didn't discriminate against each type of complication posing caution in the interpretation of the results and their generalisability. Third, a high degree of heterogeneity in terms of the surgical techniques, grafts used, different type of autograft and different type of synthetic grafts, and a higher percentage of male patients was found. In this study, we have not taken into account the autograft of the quadriceps tendon which is an emerging technique and for that reason, there are no comparative studies in the literature with a tangible case to be inserted. It should also be considered that the percentage of BPTB was low, and it was not possible to perform an analysis of the results based on the type of autograft used for all of the outcome measures considered. Finally, we included studies with different evaluation times; it is likely that both PROMs and complication rates are affected by the length of patient follow‐up, thereby suggesting that the outcomes could also be potentially different between the two procedures if a specific follow‐up time was determined. Nonetheless, the major methodological strengths of this study are the comparative nature of the article inclusion strategy and the pooling of effect sizes to identify differences between the two fixation methods.

This study should serve as an update of current evidence on outcomes of ACLR according to the graft used. The meta‐analysis includes the largest number of comparative studies on the subject to date and analyses functional outcomes, knee stability and the rates of complications and survival. Findings of the current study suggest that ACLR with synthetic graft reported a statistically but not clinically greater knee stability; moreover, it should be considered that these results are valid for the subset of grafts analysed in this study, with a higher percentage of HT compared to BPTB grafts.

## CONCLUSIONS

This meta‐analysis of comparative studies showed that compared to ACLR with HT autograft, the ACLR with synthetic graft showed statistically but not clinically important decreased knee laxity. Comparable functional outcomes, complication and re‐rupture rates were found between the two groups. Future studies including a large number of patients, using the same surgical technique, and being evaluated in long‐term randomised‐controlled trials should be conducted to confirm these findings and allow surgeons to choose the better graft optimising outcomes of ACLR.

## AUTHOR CONTRIBUTIONS

Michele Mercurio conceived and designed the study, performed the statistical analysis, participated in the acquisition and interpretation of data, and drafted the manuscript. Erminia Cofano participated in the acquisition and interpretation of data, performed the statistical analysis, and drafted the manuscript. Orlando Cosentino participated in the acquisition and interpretation of data and drafted the manuscript. Katia Corona participated in the acquisition and interpretation of data and drafted the manuscript. Fabrizio Mocini participated in the interpretation of data and drafted the manuscript. Umberto Rossi participated in the acquisition and interpretation of data and drafted the manuscript. Olimpio Galasso conceived and coordinated the study and revised critically the manuscript. Giorgio Gasparini conceived and coordinated the study and revised critically the manuscript, Simone Cerciello conceived and coordinated the study, drafted the manuscript and approved the final version of the manuscript as submitted. All authors have read and approved the final manuscript, and all authors believe that the manuscript represents honest work.

## CONFLICT OF INTEREST STATEMENT

The authors declare no conflicts of interest.

## ETHICS STATEMENT

Not applicable.

## Supporting information


**Supplementary Table 1:** Quality assessment of included studies according to the Modified Newcastle‐Ottawa scale. **Supplementary Table 2:** Risk of bias graph: review authors' judgements about each risk of bias item presented as percentages across all included studies. **Supplementary Table 3**: Risk of bias summary: review authors' judgements about each risk of bias item for each included study. **Supplementary Materials 1:** Comparison of mean age between autograft and synthetic graft reconstruction groups: forest plot of effect sizes. **Supplementary Materials 2:** Comparison of the time from injury to surgery between autograft and synthetic graft reconstruction groups: forest plot of effect sizes. **Supplementary Materials 3:** Comparison of the time from injury to surgery between hamstring autograft and synthetic graft reconstruction groups: forest plot of effect sizes. **Supplementary Materials 4**: Comparison of the time to surgery between BPTB autograft and synthetic graft reconstruction groups: forest plot of effect sizes. **Supplementary Materials 5:** Comparison of the preoperative Lysholm activity scale between autograft and synthetic graft reconstruction groups: forest plot of effect sizes. **Supplementary Materials 6:** Comparison of the preoperative Lysholm activity scale between hamstring autograft and synthetic graft reconstruction groups: forest plot of effect sizes. **Supplementary Materials 7**: Comparison of the preoperative Lysholm activity scale between BPTB autograft and synthetic graft reconstruction groups: forest plot of effect sizes. **Supplementary Materials 8:** Comparison of the preoperative Tegner activity scale between autograft and synthetic graft reconstruction groups: forest plot of effect sizes. **Supplementary Materials 9:** Comparison of the preoperative Tegner activity scale between hamstring autograft and synthetic graft reconstruction groups: forest plot of effect sizes. **Supplementary Materials 10:** Comparison of the preoperative IKCD score between autograft and synthetic graft reconstruction groups: forest plot of effect sizes. **Supplementary Materials 11:** Comparison of the preoperative IKCD score between hamstring autograft and synthetic graft reconstruction groups: forest plot of effect sizes. **Supplementary Materials 12:** Comparison of the postoperative Lysholm activity scale between hamstring autograft and synthetic graft reconstruction groups: forest plot of effect sizes. **Supplementary Materials 13:** Comparison of the postoperative Lysholm activity scale between BPTB autograft and synthetic graft reconstruction groups: forest plot of effect sizes. **Supplementary Materials 14:** Comparison of the postoperative Tegner activity scale between hamstring autograft and synthetic graft reconstruction groups: forest plot of effect sizes. **Supplementary Materials 15**: Comparison of the postoperative Tegner activity scale between BPTB autograft and synthetic graft reconstruction groups: forest plot of effect sizes. **Supplementary Materials 16:** Comparison of the postoperative IKCD score between hamstring autograft and synthetic graft reconstruction groups: forest plot of effect sizes. **Supplementary Materials 17:** Comparison of the overall complications rate between hamstring autograft and synthetic graft reconstruction groups: forest plot of effect sizes. **Supplementary Materials 18:** Comparison of the overall complications rate between BPTB autograft and synthetic graft reconstruction groups: forest plot of effect sizes. **Supplementary Materials 19:** Comparison of the re‐rupture rate between hamstring autograft and synthetic graft reconstruction groups: forest plot of effect sizes. **Supplementary Materials 20:** Comparison of the re‐rupture rate between BPTB autograft and synthetic graft reconstruction groups: forest plot of effect sizes. **Supplementary Materials 21**: Comparison of the re‐intervention rate between hamstring autograft and synthetic graft reconstruction groups: forest plot of effect sizes. **Supplementary Materials 22:** Comparison of the re‐intervention rate between BPTB autograft and synthetic graft reconstruction groups: forest plot of effect sizes.

## Data Availability

Data sharing is not applicable to this article as no datasets were generated or analysed during the current study.
