# Supplementary material for: Hamstring autograft, bone‐patellar‐tendon‐bone autograft and synthetic graft in primary anterior cruciate ligament reconstruction: A meta‐analysis of comparative studies
Source: J Exp Orthop. 2025 Jul 18;12(3):e70326. doi: 10.1002/jeo2.70326 (PMC12272514; doi:10.1002/jeo2.70326)
Supplement: Supplementary file 1 — Supplementary Table 1: Quality assessment of included studies according to the Modified Newcastle‐Ottawa scale. Supplementary Table 2: Risk of bias graph: review authors' judgements about each risk of bias item presented as percentages across all included studies. Supplementary Table 3: Risk of bias summary: review authors' judgements about each risk of bias item for each included study. Supplementary Materials 1: Comparison of mean age between autograft and synthetic graft reconstruction groups: forest plot of effect sizes. Supplementary Materials 2: Comparison of the time from injury to surgery between autograft and synthetic graft reconstruction groups: forest plot of effect sizes. Supplementary Materials 3: Comparison of the time from injury to surgery between hamstring autograft and synthetic graft reconstruction groups: forest plot of effect sizes. Supplementary Materials 4: Comparison of the time to surgery between BPTB autograft and synthetic graft reconstruction groups: forest plot of effect sizes. Supplementary Materials 5: Comparison of the preoperative Lysholm activity scale between autograft and synthetic graft reconstruction groups: forest plot of effect sizes. Supplementary Materials 6: Comparison of the preoperative Lysholm activity scale between hamstring autograft and synthetic graft reconstruction groups: forest plot of effect sizes. Supplementary Materials 7: Comparison of the preoperative Lysholm activity scale between BPTB autograft and synthetic graft reconstruction groups: forest plot of effect sizes. Supplementary Materials 8: Comparison of the preoperative Tegner activity scale between autograft and synthetic graft reconstruction groups: forest plot of effect sizes. Supplementary Materials 9: Comparison of the preoperative Tegner activity scale between hamstring autograft and synthetic graft reconstruction groups: forest plot of effect sizes. Supplementary Materials 10: Comparison of the preoperative IKCD score between autograft and synthet [file JEO2-12-e70326-s001.docx]

| **Study Author (year)** | **Criteria** | | | | | | | | **Total** | **Quality** |
| --- | --- | --- | --- | --- | --- | --- | --- | --- | --- | --- |
|  | **1** | **2** | **3** | **4** | **5** | **6** | **7** | **8** |  |  |
| Bianchi et al. (2018) | 1 | 1 | 1 | 1 | 2 | 1 | 1 | 1 | 9 | High |
| Chen et al. (2017) | 1 | 1 | 1 | 1 | 2 | 1 | 1 | 1 | 9 | High |
| Hamido et al. (2015) | 1 | 1 | 1 | 1 | 2 | 1 | 1 | 1 | 9 | High |
| Liu et al. (2009) | 1 | 1 | 1 | 1 | 2 | 1 | 1 | 1 | 9 | High |
| Moretti et al. (2023) | 1 | 1 | 1 | 1 | 2 | 1 | 1 | 1 | 9 | High |
| Pan et al. (2012) | 1 | 1 | 1 | 1 | 2 | 1 | 1 | 1 | 9 | High |
| Pritchett (2012) | 1 | 1 | 1 | 1 | 2 | 1 | 1 | 1 | 9 | High |
| Su et al. (2018) | 1 | 1 | 1 | 1 | 2 | 1 | 1 | 1 | 9 | High |

Based on the total score, quality was classified as “low” (0-3), “moderate” (4-6) and “high” (7-9).Criterion number (in bold): 1, representativeness of the exposed cohort; 2, selection of the nonexposed cohort; 3, ascertainment of exposure; 4, demonstration that outcome of interest was not present at start of study; 5, comparability of cohorts on the basis of the design or analysis; 6, assessment of outcome; 7, was follow-up long enough for outcomes to occur?; 8, adequacy of follow up of cohorts. Each study was awarded a maximum of one or two points for each numbered item within categories, based on the Modified Newcastle-Ottawa scale rules*.*

**Supplementary Table 1** Quality assessment of included studies according to the Modified Newcastle-Ottawa scale


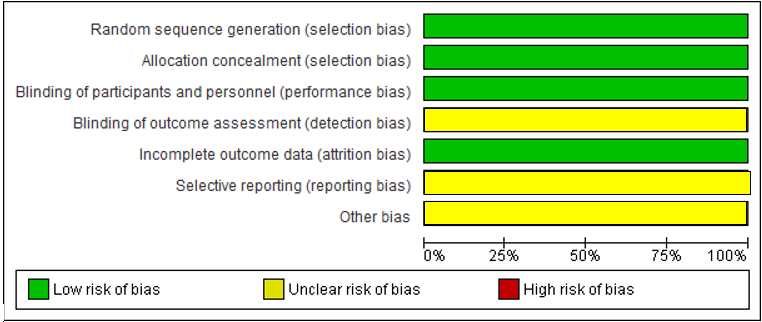


**Supplementary Table 2**. Risk of bias graph: review authors' judgements about each risk of bias item presented as percentages across all included studies.


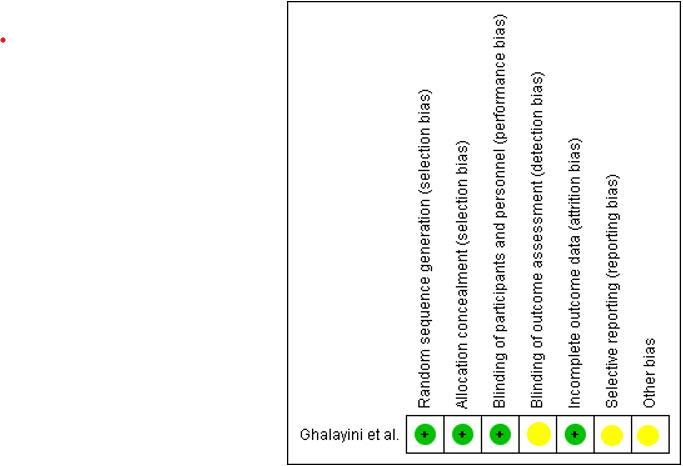


**Supplementary Table 3.** Risk of bias summary: review authors' judgements about each risk of bias item for each included study.


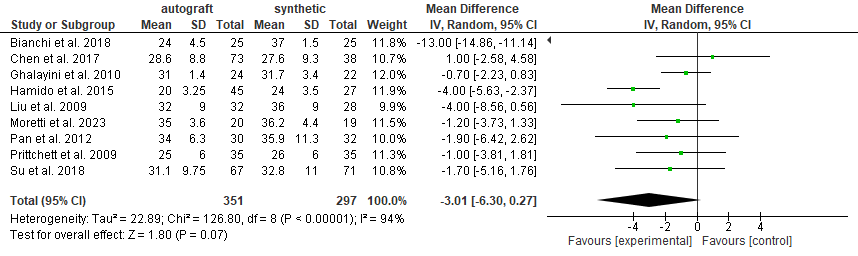


**Supplementary Material 1**. Comparison of mean age between autograft and synthetic graft reconstruction groups: forest plot of effect sizes


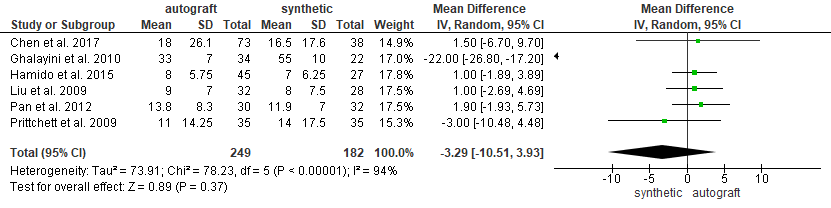


**Supplementary Material 2.** Comparison of the time from injury to surgery between autograft and synthetic graft reconstruction groups: forest plot of effect sizes

**
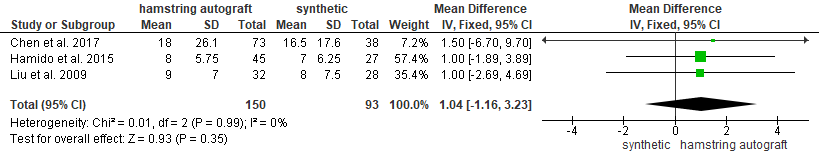
**

**Supplementary Material 3.** Comparison of the time from injury to surgery between hamstring autograft and synthetic graft reconstruction groups: forest plot of effect sizes


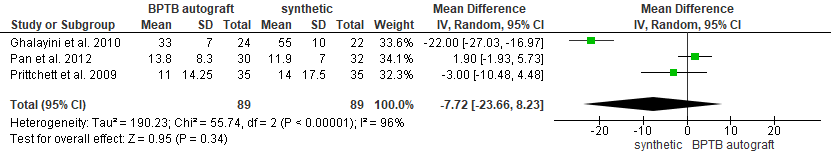
 **Supplementary Material 4.** Comparison of the time to surgery between BPTB autograft and synthetic graft reconstruction groups: forest plot of effect sizes


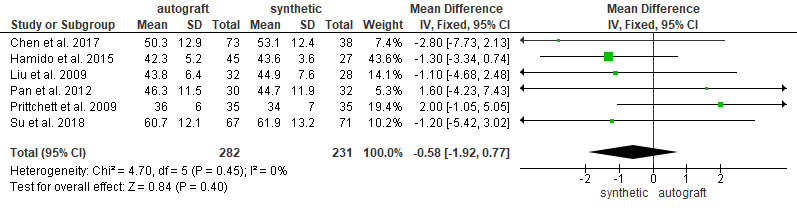


**Supplementary Material 5**. Comparison of the preoperative Lysholm activity scale between autograft and synthetic graft reconstruction groups: forest plot of effect sizes

**
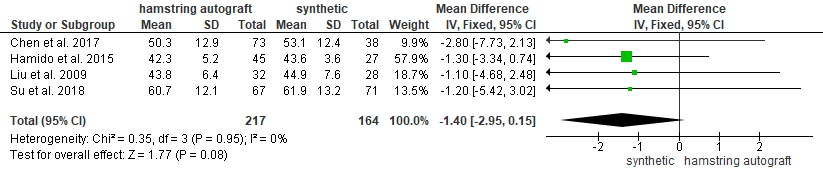
**

**Supplementary Material 6.** Comparison of the preoperative Lysholm activity scale between hamstring autograft and synthetic graft reconstruction groups: forest plot of effect sizes


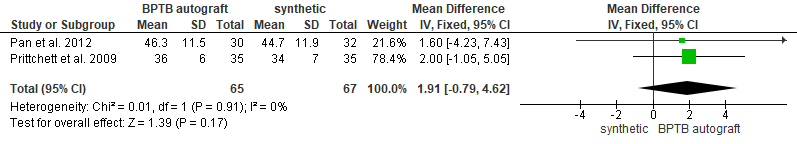


**Supplementary Material 7.** Comparison of the preoperative Lysholm activity scale between BPTB autograft and synthetic graft reconstruction groups: forest plot of effect sizes


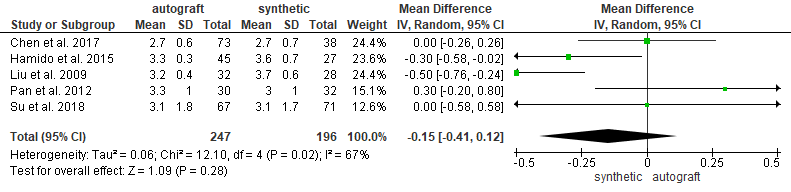


**Supplementary Material 8.** Comparison of the preoperative Tegner activity scale between autograft and synthetic graft reconstruction groups: forest plot of effect sizes


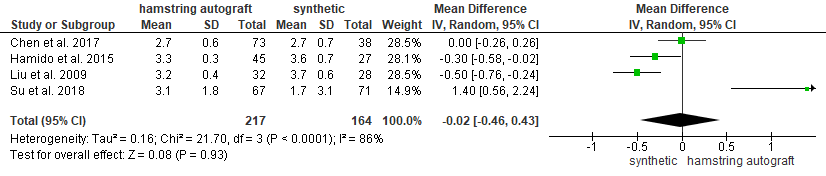


**Supplementary Material 9**. Comparison of the preoperative Tegner activity scale between hamstring autograft and synthetic graft reconstruction groups: forest plot of effect sizes


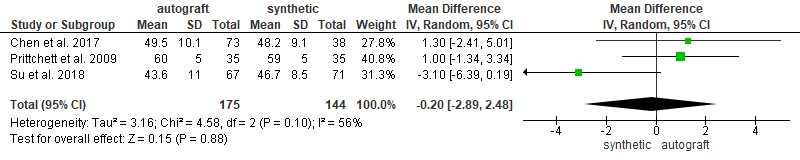


**Supplementary Material 10**. Comparison of the preoperative IKCD score between autograft and synthetic graft reconstruction groups: forest plot of effect sizes


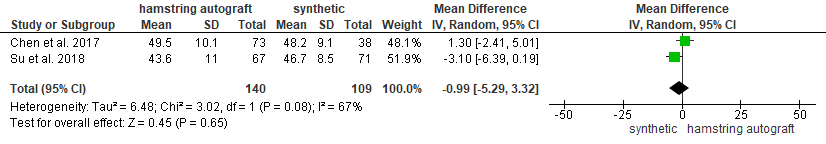


**Supplementary Material 11**. Comparison of the preoperative IKCD score between hamstring autograft and synthetic graft reconstruction groups: forest plot of effect sizes


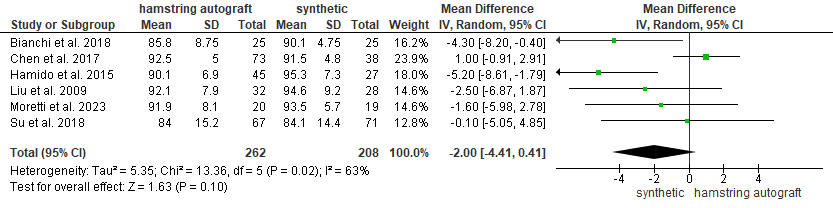


**Supplementary Material 12**. Comparison of the postoperative Lysholm activity scale between hamstring autograft and synthetic graft reconstruction groups: forest plot of effect sizes

**
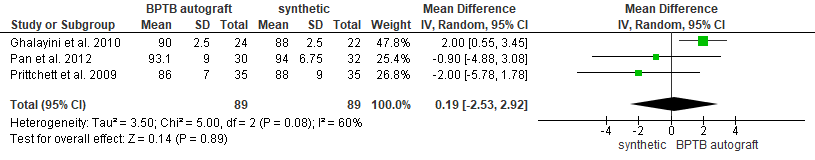
**

**Supplementary Material 13.** Comparison of the postoperative Lysholm activity scale between BPTB autograft and synthetic graft reconstruction groups: forest plot of effect sizes


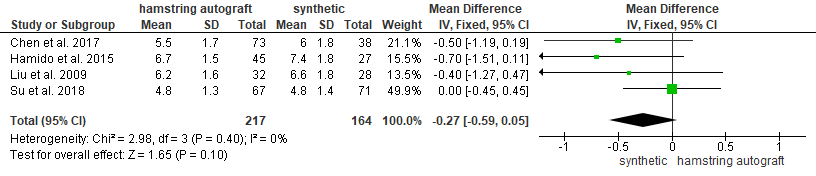


**Supplementary Material 14.** Comparison of the postoperative Tegner activity scale between hamstring autograft and synthetic graft reconstruction groups: forest plot of effect sizes

**
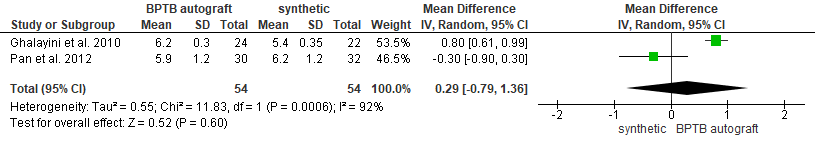
**

**Supplementary Material 15.** Comparison of the postoperative Tegner activity scale between BPTB autograft and synthetic graft reconstruction groups: forest plot of effect sizes


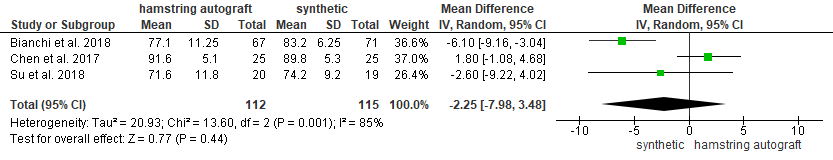


**Supplementary Material 16.** Comparison of the postoperative IKCD score between hamstring autograft and synthetic graft reconstruction groups: forest plot of effect sizes


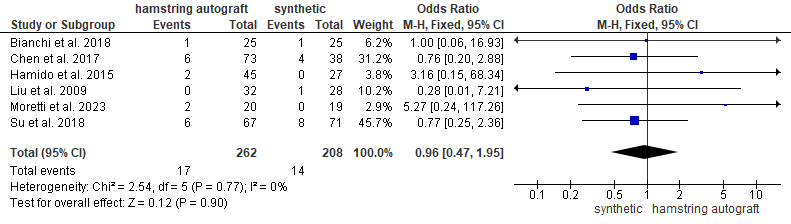


**Supplementary Material 17.** Comparison of the overall complications rate between hamstring autograft and synthetic graft reconstruction groups: forest plot of effect sizes


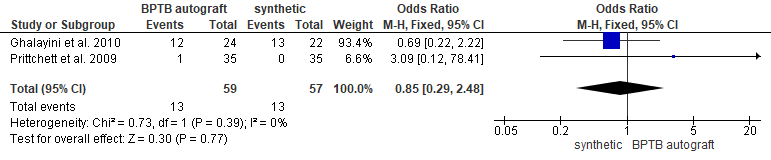


**Supplementary Material 18**. Comparison of the overall complications rate between BPTB autograft and synthetic graft reconstruction groups: forest plot of effect sizes


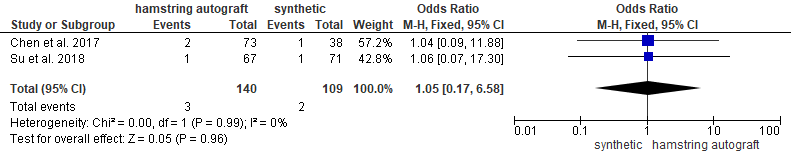


**Supplementary Material 19.** Comparison of the re-rupture rate between hamstring autograft and synthetic graft reconstruction groups: forest plot of effect sizes


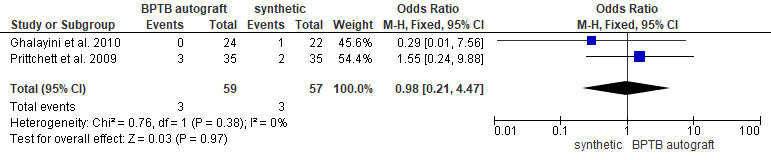


**Supplementary Material 20**. Comparison of the re-rupture rate between BPTB autograft and synthetic graft reconstruction groups: forest plot of effect sizes


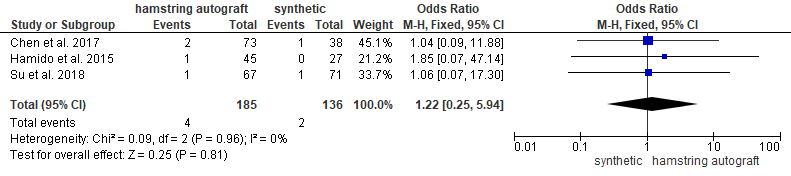


**Supplementary Material 21**. Comparison of the re-intervention rate between hamstring autograft and synthetic graft reconstruction groups: forest plot of effect sizes


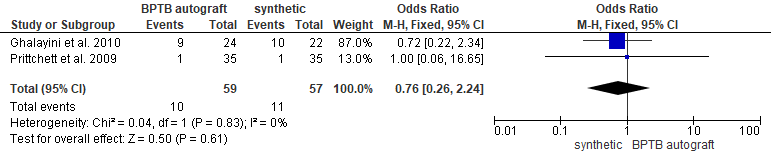


**Supplementary Material 22**. Comparison of the re-intervention rate between BPTB autograft and synthetic graft reconstruction groups: forest plot of effect sizes
